# The renal phenotype of allopurinol-treated HPRT-deficient mouse

**DOI:** 10.1371/journal.pone.0173512

**Published:** 2017-03-10

**Authors:** Cristina Zennaro, Federica Tonon, Paola Zarattini, Milan Clai, Alessandro Corbelli, Michele Carraro, Marialaura Marchetti, Luca Ronda, Gianluca Paredi, Maria Pia Rastaldi, Riccardo Percudani

**Affiliations:** 1 Department of Medical, Surgery and Health Sciences, Università degli Studi di Trieste, Trieste, Italy; 2 Department of Life Sciences, Università degli Studi di Trieste, Trieste, Trieste, Italy; 3 Department of Pathology and Legal Medicine, Azienda Sanitaria Universitaria Integrata di Trieste, Trieste, Italy; 4 Unit of Bio-imaging, Department of Cardiovascular Research, IRCCS Mario Negri Institute for Pharmacological Research, Milano, Italy; 5 Department of Life Sciences, University of Parma, Parma, Italy; 6 Department of Neurosciences, University of Parma, Parma, Italy; 7 Department of Pharmacy and SITEIA, PARMA Interdepartmental Center, University of Parma, Parma, Italy; 8 Fondazione IRCCS Ca’ Granda Ospedale Maggiore Policlinico, Milano, Italy; UCL Institute of Child Health, UNITED KINGDOM

## Abstract

Excess of uric acid is mainly treated with xanthine oxidase (XO) inhibitors, also called uricostatics because they block the conversion of hypoxanthine and xanthine into urate. Normally, accumulation of upstream metabolites is prevented by the hypoxanthine-guanine phosphoribosyltransferase (HPRT) enzyme. The recycling pathway, however, is impaired in the presence of HPRT deficiency, as observed in Lesch-Nyhan disease. To gain insights into the consequences of purine accumulation with HPRT deficiency, we investigated the effects of the XO inhibitor allopurinol in Hprt-lacking (HPRT^-/-^) mice. Allopurinol was administered in the drinking water of E12-E14 pregnant mothers at dosages of 150 or 75 μg/ml, and mice sacrificed after weaning. The drug was well tolerated by wild-type animals and heterozygous HPRT^+/-^ mice. Instead, a profound alteration of the renal function was observed in the HPRT^-/-^ model. Increased hypoxanthine and xanthine concentrations were found in the blood. The kidneys showed a yellowish appearance, diffuse interstitial nephritis, with dilated tubules, inflammatory and fibrotic changes of the interstitium. There were numerous xanthine tubular crystals, as determined by HPLC analysis. Oil red O staining demonstrated lipid accumulation in the same location of xanthine deposits. mRNA analysis showed increased expression of adipogenesis-related molecules as well as profibrotic and proinflammatory pathways. Immunostaining showed numerous monocyte-macrophages and overexpression of alpha-smooth muscle actin in the tubulointerstitium. *In vitro*, addition of xanthine to tubular cells caused diffuse oil red O positivity and modification of the cell phenotype, with loss of epithelial features and appearance of mesenchymal characteristics, similarly to what was observed *in vivo*. Our results indicate that in the absence of HPRT, blockade of XO by allopurinol causes rapidly developing renal failure due to xanthine deposition within the mouse kidney. Xanthine seems to be directly involved in promoting lipid accumulation and subsequent phenotype changes of tubular cells, with activation of inflammation and fibrosis.

## Introduction

Lesch-Nyhan disease (LND) is a rare X-linked monogenic disorder caused by an inborn error of nucleotide metabolism. The mutations are localized on the gene *HPRT1*, which encodes the enzyme hypoxanthine-guanine phosphoribosyltransferase, a key molecule in recycling purine bases. Being an X-linked recessive disorder, the disease occurs almost entirely in males; occurrence in females is extremely rare [1]. A direct consequence of HPRT deficiency is the augmented degradation of free purines and results in overproduction of the end-product uric acid, which accumulates in plasma (hyperuricemia) and urine (hyperuricosuria).

Hyperuricemia leads to precipitation of uric acid crystals in certain areas of the body, particularly in the joints. Hyperuricosuria leads to formation of sandy sludge or stones in the urinary collecting system, causing renal failure if not promptly treated [1]. Though some advances have been made in recent years, the treatment of choice remains allopurinol, which has demonstrated efficacy in controlling the overproduction of uric acid in LND patients. However, the drug blocks the enzyme xanthine oxidase (XDH), thereby increasing the upstream formation of oxypurines, i.e. xanthine and hypoxanthine, which can be harmful to cell metabolism [2].

In addition, LND patients exhibit characteristic neurological disorders; they suffer from a severe motor handicap associated with generalized dystonia. Most of them have cognitive disability, often not severe. Patients also show an unusual tendency towards pathologic behavior, including self-injury, with self-biting being particularly prominent [1]. Despite many research efforts, the pathogenesis of this neurobehavioral syndrome remains poorly understood. In these patients, the brain appears structurally normal. A dysfunction of the basal ganglia has been suggested, with dopamine neurons adversely affected and a significant reduction of dopamine secretion [3, 4].

Mouse studies have mainly profited from the generation of HPRT-deficient mice (HPRT^-/-^) [5], which exhibit some of the metabolic abnormalities of LND, including loss of Hprt enzyme activity, failure in purine recycling, and consequently accelerated synthesis of purines. However, these mice do not have hyperuricemia and obvious neurobehavioral abnormalities [1]. One possible explanation for the absence of a clear LND phenotype in HPRT^-/-^ animals has been ascribed to the presence of enzyme urate oxidase (UOX), which converts uric acid to the soluble allantoin that is absent in humans due to evolutionary inactivation [6]. However, several clinical studies point to a potential neuroprotective role of uric acid and have demonstrated a robust inverse correlation between uric acid levels and the clinical progression of neurological disorders, such as Parkinson's disease [7, 8]. Also, allopurinol treatment in humans is not successful against the neurological symptoms of LND patients, despite the return to normal uricemic levels [9].

Some *in vitro* analyses support the hypothesis that HPRT deficiency *per se* alters the transcriptional activity of dopaminergic neurons, resulting in loss of dopamine and functional damage of the basal ganglia [10], but the absence of neurobehavioral symptoms in HPRT^-/-^ mice seems to contradict this possibility.

As allopurinol is administered to LND patients as soon as possible after birth, we hypothesized that allopurinol treated HPRT^-/-^ mice could represent a better model of the human disease and provide insights into the consequences of free purine accumulation in the presence of HPRT-deficiency.

Therefore, we decided to administer allopurinol to HPRT^-/-^, HPRT^+/-^, and *wild type* mice. The drug was added to the drinking water of pregnant mothers at E12-E14 at dosages based on those utilized in UOX^-/-^ mice [6] to prevent uric acid deposition and death by renal failure.

## Materials and methods

### Animals and allopurinol treatment

The HPRT-deficient strain (B6.129P2-Hprtb-m3/J) was purchased from The Jackson Laboratory (Sacramento, CA, US) and maintained according to the Animal Welfare Guidelines.

This study was carried out in strict accordance with the recommendations in the Guide for the Care and Use of Laboratory Animals of the National Institutes of Health. The animal protocol was approved by the Italian Ministry of Health (authorization number 625/2015, released on July 3, 2015) in compliance with Italian regulation (D.L. vo 26/2014). All efforts were made to minimize suffering in all procedures and the sacrifice was performed by cervical dislocation.

Breeding conditions were the following: temperature 22–25°C; relative humidity 45–55%; ventilation 10–15 complete air changes per hour; light/dark cycle 12h/12h; pellet food (Envigo Teklad 2018, Envigo Rms s.r.l, San Pietro al Natisone, Italy) and water ad libitum.

Mice were treated starting from embryonic day 12–14 with allopurinol (Sigma-Aldrich Co., St Louis, MO, USA) or vehicle administered continuously in the drinking water to pregnant females[6], and was continued after birth until sacrifice. Allopurinol was dissolved in 1 N NaOH at 100 mg/ml concentration and then diluted in the drinking water to the final concentration of 75 μg/ml.

Mice were sacrificed at 1 or 2 months of age. Urine samples were collected from the bladder and examined by phase contrast and/or stereo microscopy (Leica Microsystems, Wetzlar, Germany). After blood collection, serum was separated and stored at -80°C.

### Blood values

Blood urea nitrogen (BUN) and xanthine oxidase activity were determined using commercial kits according to the manufacturer indications (Sigma-Aldrich Co.). Serum creatinine was measured by HPLC method. Briefly 12.5 μl of sera were deproteinized by adding 1 ml of 100% acetonitrile. Samples were then vortexed and centrifuged twice for 20 minutes at 4°C, 16000 g. After vacuum evaporation of the supernatant fraction, dried samples were suspended in 12.5 μl of 5 mM NaH_2_PO_4_, pH 6.4 (mobile phase) and stored at -20°C. Samples were manually injected by a 10 μl loop on a Prominence HPLC system (Shimadzu, Kyoto, Japan). Analytes were separated on a SCX column (Zorbax SCX-300 4.6 x 250, Agilent Technologies, Santa Clara, CA, US) with an isocratic run and revealed at 234 nm and 240 nm. Data were collected and analyzed by LabSolutions software. The output peaks where identified and quantified by comparison with chromatographic runs of creatinine standards (Sigma-Aldrich).

### Purine HPLC analysis of mouse sera

Mouse sera obtained by centrifugation of the whole blood were stored at -80°C until needed. Deproteinization was performed by adding 0.4 M perchloric acid at the ratio 1:1.67 (v/v) [11]. Deproteinized samples were incubated on ice overnight and then centrifuged twice for 20 minutes at 4°C, 16000 g. The supernatants were transferred in new tubes, and finally stored at -20°C. Samples (10 μl) were injected on a HPLC system (Prominence Shimadzu, Kyoto, Japan). Analytes were separated on a reversed phase C18 column (Microsorb-MV 100–5 C18 150x4.6 mm, Agilent Technologies, Santa Clara, CA, US) and revealed at 254 nm and 270 nm. Chromatographic runs were carried out with a dual mobile phases gradient [11]. The mobile phase A contained 0.5 mM sodium pentanesulfonate and 10 mM KH_2_PO_4_, pH 3.5 and the mobile phase B has the same composition of phase A in 10% acetonitrile (v/v). The output peaks where identified and quantified by comparison with chromatographic runs of purine standards (Sigma Aldrich Co).

### Renal examination

Kidneys were taken and immediately dissected for differential processing. Formalin-fixed samples were dehydrated and paraffin-embedded. 2–3 μm thick sections were cut using a Microm HM450 sliding microtome and stained with hematoxylin&eosin, PASand reticulin fibers impregnation (Gordon Sweet, GW), using commercial kits (Bio Optica Milano S.p.A, Italy).

The presence of collagen fibers was detected applying the Picrosirus-Red (PR) and Masson's Trichrome (MT) methods. Briefly, paraffin-embedded sections were dehydrated and stained with Sirius Red (BDH Chemical Ltd Poole England) 1 mg/ml dissolved in Picric Acid-Saturated Solution 1.3% (Sigma-Aldrich Co.), then rehydrated and mounted with aqueous mounting medium. For the Masson's Trichrome staining we used a commercial kit (DAKO Italia S.r.l, Italy).

Quantification of interstitial fibrosis was performed on GW, MT and PR sections from 5 animals per genotype by calculating the percentage of area occupied by the specific staining in 10 fields/section, examined at 200X magnification. Images were recorded by a high-resolution video camera (Q-Imaging Fast 1394), and quantified by the image analysis software Image-Pro^®^Plus (vers.6.3, Media-Cybernetics; Silver Spring, MD, USA).

For crystals evaluation, a piece of the kidney was fixed in absolute ethanol, then diaphanized with xylene, and paraffin-embedded.

To observe lipid deposits Oil Red O stain was conducted on tissue embedded within OCT (Bio Optica), then frozen and stored at -80°C. 5 to 8 μm thick cryo-sections were then fixed in 10% formalin, briefly washed with running tap water, immersed in 60% isopropanol, and stained with Oil Red O (Sigma-Aldrich Co.) 3 mg/ml dissolved in isopropanol, followed by alum haematoxylin. Slides were mounted with glycerol for microscope examination.

Transmission electron microscopy (TEM) was performed on 1 mm^3^ pieces of tissue fixed in a mixture of paraformaldehyde and glutaraldehyde in 120 mM phosphate buffer, post-fixed in 1% osmium tetroxide in 120 mM cacodylate buffer, dehydrated, and embedded in Epon–Araldite resin. Ultra-thin sections were cut and placed on grids, stained by uranyl-acetate and lead-citrate, and observed under an Energy Filter Transmission Electron Microscope (EFTEM, ZEISS LIBRA^®^ 120) equipped with YAG scintillator slow-scan CCD camera.

### Kidney stone analysis

Birefringent crystals were isolated from kidney slices using crystallization tools and solubilized in phosphate-buffered saline (PBS). The solution was treated with 0.4 M perchloric acid overnight to precipitate protein traces, centrifuged and concentrated before HPLC separation.

### Gene analysis

Total RNA from kidneys was extracted using Trizol (Invitrogen Molecular Probes, Carlsbad, CA, US). RNA concentrations were determined using Nanodrop ND 1000 (Euroclone S.p.A., Milan, Italy). One microgram of total RNA was reverse transcribed using MMLV reverse transcriptase (Applied Biosystems, Foster City, CA, USA). The analyses were performed by quantitative Real Time RT-PCR (QRT-PCR) utilizing SYBRGreen Master Mix buffer (Applied Biosystems). The primers utilized, according to Ohtsubo et al [12] are listed in S1 Table; GAPDH was used as house-keeping gene.

For the QRT-PCR the amplification steps were: pre-denaturation at 95°C for 10 min, 40 cycles of amplification with denaturation at 95°C for 15 s, annealing at proper temperature for 60s and extension at 72°C for 30 s. A final extension at 72°C for 10 min and a dissociation stage (95/60/95°C for 15 s each) were then added.

### Immunohistochemistry and immunofluorescence of kidney section

Immunostaining was performed on paraffin or frozen sections. Kidney sections were incubated with mouse anti-human α-SMA (1:75; DAKO, Agilent Technologies) and rat anti-mouse CD68 (1:50; SEROTEC, Bio-Rad, Hercules, CA, US), then with biotinylated (VECTOR Laboratories, Burlingame, CA, US) or FITC-conjugated (Invitrogen Molecular Probes) secondary antibodies. After immunohistochemistry, sections were counterstained with hematoxylin.

For quantitative analysis, 10 fields per section were digitally acquired at 200X magnification by a high-resolution video camera (Q-Imaging Fast 1394), and the percentage of α-SMA positive areas or the number of macrophages per field were calculated by the image analysis software Image-Pro^®^Plus (vers.6.3, Media-Cybernetics). Five animals per genotype were analyzed.

### Cell cultures

The MDCK (Madin-Darby canine kidney) cell line was cultured in MEM medium (Euroclone S.p.A.) supplemented with 10% heat inactivated Fetal Bovine Serum (Gibco, Thermo-Fisher Scientific, Waltham, MA, USA), 100 U/ml penicillin, 100 μg/ml streptomycin and 20 mM L-glutamine (Euroclone S.p.A.). The cell line was grown at 37°C in a humidified atmosphere at 5% CO_2_, seeded at densities of 1.7·10^3^/cm^2^ (96 hours of incubation) or 3.4·10^3^/cm^2^ (48 hours of incubation) in 6-well plates, and treated with 1mg/ml xanthine or 0.06 mg/ml uric acid (Sigma-Aldrich Co.) for 48 and 96 hours, then collected for western blot analysis or fixed with 4% paraformaldehyde for immunostaining.

### Cell counting and vitality assay

To evaluate the viability and number of cells that had to be sown, the Trypan blue exclusion assay was performed. This test allows direct identification and enumeration of live blue cells and, unstained dead cells in a given population. To this purpose, a cell aliquot was diluted in an equal volume of 0.04% solution of Trypan blue in 1X Phosphate-buffer saline (PBS). Then, cell counting was performed by loading 15 μl of cell re-suspension diluted 1:1 with 0.04% Trypan blu in Thoma’s counting chamber. The preparation was examined by optical microscope (Nikon Eclipse TS100; Nikon Corporation, Tokyo, Japan). After several independent cell counts, the number of live cells in 1 milliliter of medium was determined by applying the following formula: *X*_*n*_ = *n*_*m*_ * 2 * 10^4^, where X_n_ represent the cell number in 1 milliliter of medium, 2 is the dilution’s factor of Trypan blue solution and 10^4^ represent the conversion’s factor of Thoma’s counting chamber.

### Protein extraction and western blot analysis

MDCK cells were collected and lysed with the extraction buffer consisting of 45mM Tris HCl pH 6.8 (Sigma-Aldrich Co.), 0.2% N-Laurosylsarcosine (Fluka, Sigma-Aldrich Co.), 0.2 mM Phenylmethansulfonyl fluoride (PMSF, Sigma-Aldrich Co.), 1 mM 1,4-Dithiothreitol (DTT, Sigma-Aldrich Co.), protease inhibitors (2 μg/ml Aprotinin and Pepstatin; Sigma-Aldrich Co.) and phosphatase inhibitors (0.1mM Sodium Orthovanadate and Sodium Fluoride; Sigma-Aldrich Co.). Protein extracts were then quantified by the Bicinchoninic (BCA) protein assay kit (Thermo-Fisher Scientific) and 40 μg of each protein extract were loaded into a 10% SDS-PAGE gel. The primary antibodies were α-SMA (Abcam, Cambridge, UK) and GAPDH (Santa Cruz Biotechnology Inc., Dallas, TX, USA). HRP-labeled IgGs were used as secondary antibodies (Santa Cruz Biotechnology Inc.).

### MTT and cytotoxicity assay

MDCK cells were seeded at a density of 1.47x10^3^ cells/cm^2^ in 96-well plates. Cellular viability and cytotoxicity were evaluated at 48 and 96 hours after xanthine or uric acid treatments. MTT was given to cells at the final concentration of 0.5 mg/ml. After 4 hours of incubation at 37°C, cell medium was removed and the salt crystals formed inside the cells were solubilized with dimethyl sulfoxide (DMSO). Absorbance was then read at 570 nm using a spectrophotometer (Spectra Max Plus 384, Molecular Devices, Sunnyvale, CA, USA). Cellular cytotoxicity was evaluated by Lactate Dehydrogenase (LDH) assay (Bio Vision Inc., Milpitas, CA). MDCK cells were seeded as in MTT assay protocol and, at the end of the appropriate treatment, 100 μl of cellular supernatant were transferred to corresponding wells in an optical clear 96-well plate. 100 μl of reaction solution, consisting of catalyst solution and dye solution in a ratio 1:45, were then added to each well and incubated for 30 minutes at room temperature, protecting the plate from light. Absorbance was finally measured at 500 nm using a spectrophotometer (Spectra Max Plus 384, Molecular Devices). As positive control, Triton X-100 (1% final concentration) treated cells were considered, whereas free medium was used as negative control.

### Cell immunostaining

Cells were fixed in 4% paraformaldehyde/0.15% picric acid for 20 minutes at room temperature. Afterwards, cells were washed thrice with PBS and incubated with 0.5% Triton X-100 for 5 minutes, then with 6% FBS/PBS for 1 hour at room temperature and finally exposed to the primary antibody (E-cadherin 1:3200, Sigma Aldrich Co.) overnight at 4°C in a humid chamber. After three quick washes, cells were incubated with the secondary anti-rat Alexa Fluor 488 (Molecular probes) diluted 1:400, for 1 hour at room temperature in the dark. After washing, slides were mounted by Moviol Mounting solution (Fluka, Sigma-Aldrich Co.). Images were acquired with a Leica DM-2000 microscope.

### Statistical analysis

Data are presented as mean ± SEM or mean ± standard deviation and statistical significance (P value) was calculated by using Instat 2.0 and Graphpad 6.1 software applying ANOVA and T-test as appropriate. P values <0.05 were considered to be statistically significant.

## Results

### Survival and renal morphology

**Fig 1:**
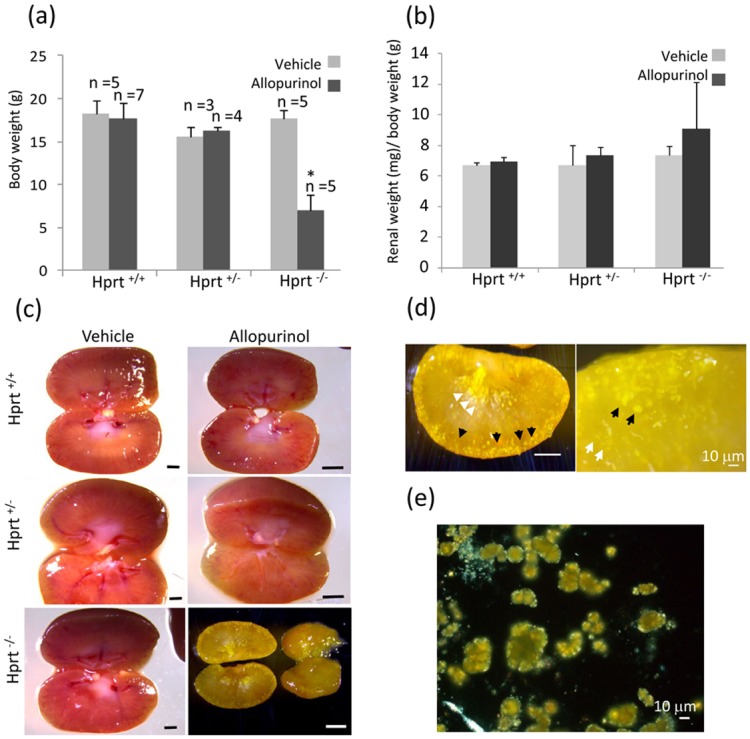
Body and kidney parameters and renal morphology. Allopurinol 75 μg/ml causes a reduction of body weight (a) and an increase of kidney/body weight ratio (b) only in KO mice. Representative macroscopic images of dissected fresh renal tissue after treatment with allopurinol 75 μg/ml or vehicle (c). Allopurinol does not cause macroscopic changes in HPRT^+/-^ and WT mice. The vehicle-treated HPRT^-/-^ kidneys have a normal appearance as well. Instead, allopurinol administered to HPRT^-/-^ animals produces profound modifications of the renal structure, with pale and yellowish appearance. This is shown in more details in panel (d), where yellow deposits can be observed in the cortex (black arrows) and yellow streaks in the medulla (white arrows). Analysis of the fresh tissue by polarized light microscopy demonstrates that the deposits are of crystalloid nature (e). *p<0.05 as compared to vehicle-treated HPRT^-/-^. Scale bars: 1 mm.

Treatment with allopurinol had highly toxic effects in HPRT^-/-^ mice, as compared to the same drug dosages administered to HPRT^+/-^ and wild type (WT) animals. All mice survived during the experimental procedures. Nonetheless, HPRT^-/-^ animals were smaller (Fig 1a and 1b) and feebler than HPRT^+/-^ and WT at age of 1 month and were sacrificed.

As compared to the other groups, allopurinol treated HPRT^-/-^ mice had increased kidney weight/body weight ratio (Fig 1b). Macroscopically, kidneys of allopurinol-treated HPRT^+/-^ and WT mice were indistinguishable from vehicle-treated animals. Instead, allopurinol-treated HPRT^-/-^ kidneys had a yellowish appearance, more evident in the cortex, but extending to the renal medulla with yellow streaks (Fig 1c and 1d). Analysis of the fresh renal tissue by polarized light microscopy demonstrated a diffuse presence of a crystalloid component (Fig 1e).

**Fig 2:**
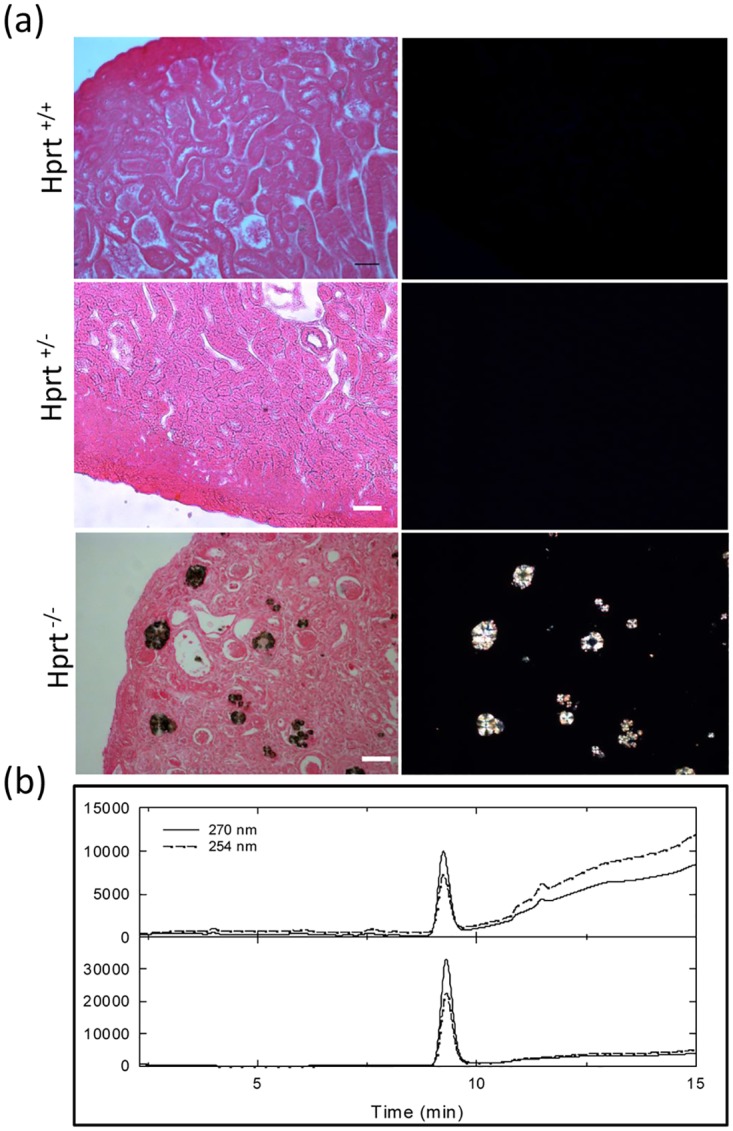
Location of crystals. (a) Representative images of ethanol-fixed eosin-stained kidney sections observed by light (left panels) and polarized microscopy (right panels). Independent of the drug dosage, renal morphology is preserved and analysis by polarized microscopy is completely negative in sections from allopurinol-treated HPRT^+/-^ and WT animals, whereas HPRT^-/-^ kidneys have altered structure with numerous crystals filling the tubular lumens. Scale bars = 50 μm. (b) Reversed-phase HPLC analysis of solubilized crystals collected from the tubular lumina of a HPRT^-/-^ kidney section showing a main peak (upper panel) identified as xanthine by comparison with a commercial standard (lower panel).

Histological analysis conducted on ethanol-fixed eosin-stained renal sections permitted to localize these deposits in profoundly altered tubules and the exam by polarized light demonstrated the crystalline nature of the deposits, which were diffusely distributed. Both tubular changes and crystals were completely absent in HPRT^+/-^ and WT animals (Fig 2a).

The crystals, isolated and dissolved from frozen renal sections, were analyzed by absorbance spectroscopy and HPLC and were demonstrated to be constituted by xanthine (Fig 2b).

**Fig 3:**
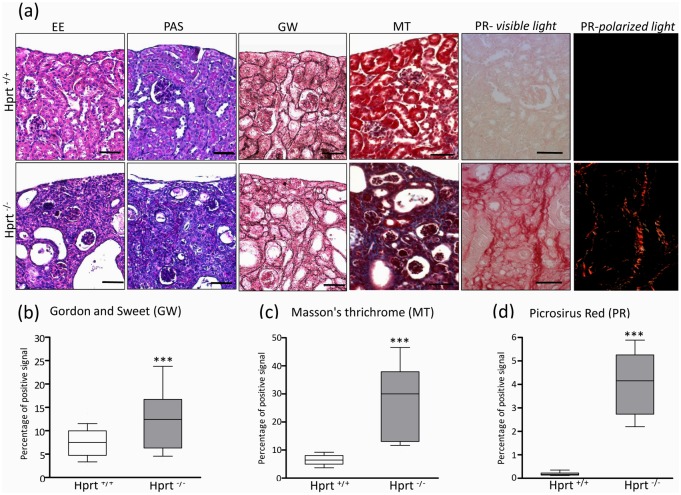
Renal histology. Histological images of kidney sections from mice treated with 75 μg/ml allopurinol (a). Normal renal morphology is detected in WT-treated mice (upper panels), whereas profound changes can be seen in HPRT^-/-^ animals (lower line of each panel), where tubules look dilated and filled by casts and cells, and abundant matrix deposition can be observed in the interstitium, where reticulin fibers are highlighted by Gordon and Sweet GW staining and collagen I/III is demonstrated PR staining. Matrix deposition affects also the glomerular structures. Scale bars = 50 μm.A statistically higher percentage of positively stained interstitial areas was observed in allopurinol-treated HPRT^-/-^ mice than WT animals, as shown in panels b (GW), c (MT), and d (PR). Data are presented as box plots, where the boxes represent the 25th to 75th percentiles, the lines within the boxes represent the median, and the lines outside the boxes represent the 10th and 90th percentiles. Results are expressed as mean ± SE. ***p< 0.001 versus HPRT^+/+^. Abbreviations: EE, hematoxylin eosin; PAS, periodic acid-schiff; GW, Gordon and Sweetstaining; MT, Masson's Trichrome, PR, Picrosirus-red.

Light microscopy examination showed completely normal morphology of allopurinol-treated HPRT^+/-^ (data non shown) and WT mice, whereas extensive renal damage was found in allopurinol-treated HPRT^-/-^ mice (Fig 3). Tubuli were dilated, with signs of tubular cell necrosis and atrophy. Inflammatory cells were present in multiple locations, sparsely distributed in the tubulointerstitium and present in the tubular lumens surrounding the crystals. Masson's thrichrome, Gordon Sweet and Picrosirius Red staining highlighted the severe degree of interstitial fibrosis due to diffuse collagen deposition (Fig 3a–3d). Though the severity of lesions mainly affected the tubulointerstitium, glomeruli were not spared and showed signs of glomerulosclerosis and increased thickness of the Bowman’s capsule (Fig 3a).

**Fig 4:**
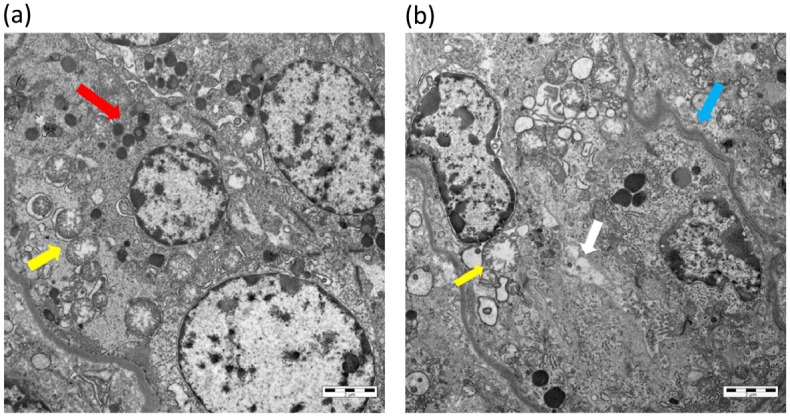
Transmission electron microscopy. Tubules from allopurinol-treated HPRT^-/-^ animals appear altered at the subcellular level, showing mitochondrial swelling (yellow arrows, a, b), numerous lysosomes (red arrow, a), shrinking of the tubular basement membrane (blue arrow, b), and loss of the brush border (white arrow, b). Scale bar = 2 μm.

Severe damage of tubular cells was confirmed at the subcellular level by transmission electron microscopy, with mitochondrial swelling, shrinkage of the tubular basement membrane, numerous lysosomes, and loss of the brush border of proximal tubular cells (Fig 4a and 4b).

### Urine and blood analyses

**Fig 5:**
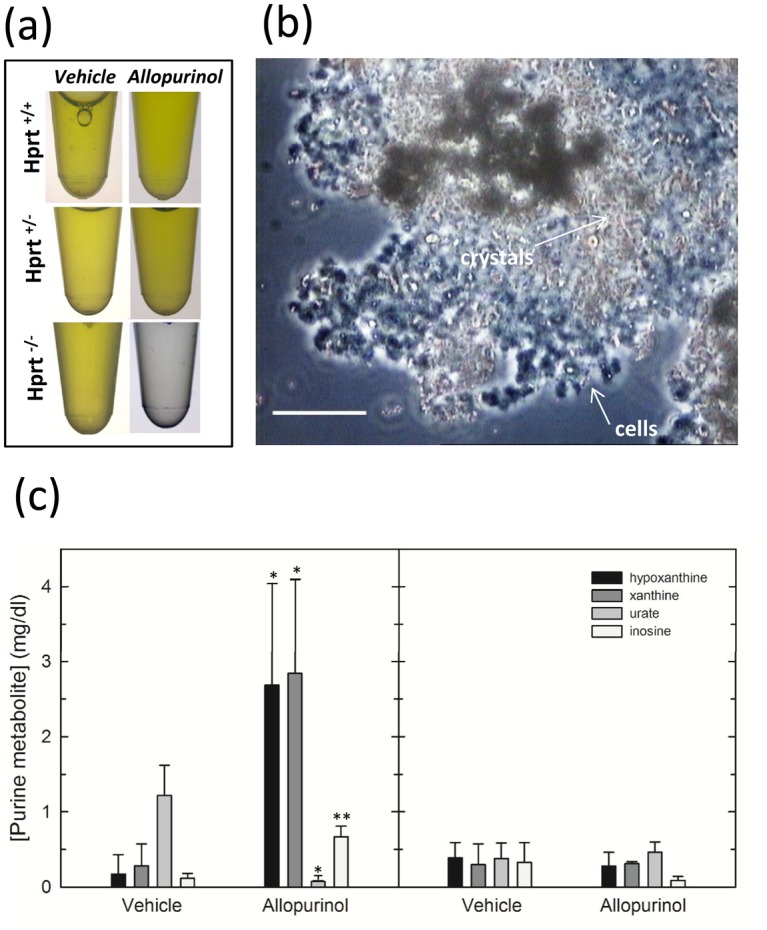
Renal function evaluation and oxidized purines /urate/inosine serum concentration. (a) Macroscopic examination of collected urine shows that, as compared to the yellow color of urine characterizing all vehicle-treated animals and allopurinol-treated WT and HPRT^+/-^ mice, a transparent urine is produced by HPRT^-/-^ mice after allopurinol administration. (b) The image is an example of the urinary sediment from an allopurinol-treated HPRT^-/-^ mouse, showing numerous cells and crystals. Scale bars = 50 μm. (c) Reversed phase HPLC analysis of blood concentration of hypoxanthine (black), xanthine (gray), urate (light gray), and inosine (white) in HPRT^-/-^ (left panel) and WT (right panel) mice. Error bars are standard deviation of the mean. *p < 0.05; **p<0.01 versus vehicle.

As compared to the yellow color of urine collected from vehicle-treated mice and allopurinol-treated HPRT^+/-^ and WT animals, the urine of allopurinol-treated HPRT^-/-^ mice appeared transparent (Fig 5a) and microscopic examination of the urinary sediment revealed the presence of numerous cells and crystals (Fig 5b).

High levels of BUN and serum creatinine were found in all HPRT^-/-^ mice treated with allopurinol (S1 Fig), confirming that these animals were affected by severe renal failure, whereas vehicle-treated mice, as well as allopurinol-treated HPRT^+/-^ and WT animals, had normal renal function.

Analysis of blood samples of allopurinol-treated HPRT^-/-^ mice by HPLC showed the accumulation of increasing concentration of xanthine, hypoxanthine, and inosine along with decreased concentration of uric acid (Fig 5c, left panel). Conversely, WT mice showed no significant changes (Fig 5c, right panel).

### The involvement of lipids

**Fig 6:**
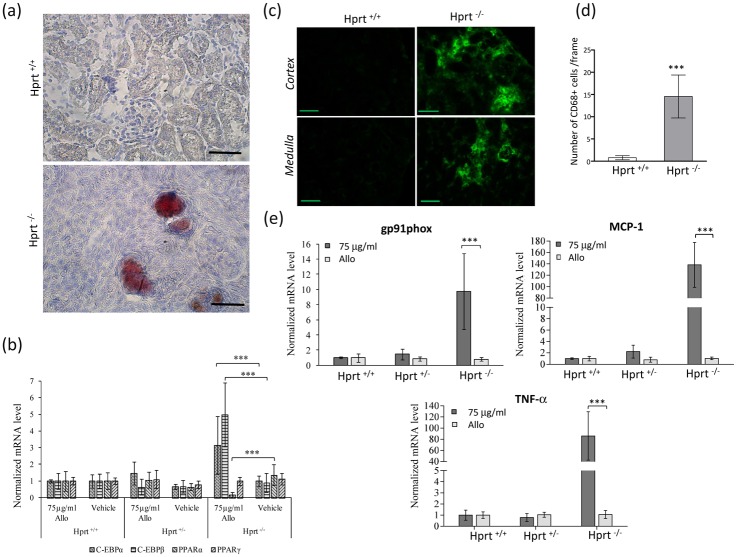
Renal lipids and inflammatory changes in kidney of HPRT^-/-^ treated mice. (a) Red Oil O staining images from allopurinol-treated WT and HPRT^-/-^ animals. The WT tissue is completely negative, whereas kidneys from HPRT^-/-^ mice show numerous positive areas in the interstitium, mainly within and around the lumen of dilated tubules. Scale bars = 50 μm. (b) Gene expression analysis of adipogenesis-related molecules C-EBP alpha and beta and PPAR alpha and gamma shows no differences based on the genotype in vehicle-treated mice, whereas a significant increase of C-EBP alpha and beta and a significant decrease of PPAR alpha are detected in allopurinol-treated HPRT^-/-^ animals. Results are expressed as mean ± standard deviation. ***p< 0.001 versus HPRT^-/-^ treated with vehicle alone. (c) The macrophage marker CD68 is present in numerous areas of the cortex and the medulla in kidney sections of allopurinol-treated HPRT^-/-^ mice (right panels), whereas it appears almost completely negative in corresponding sections of allopurinol-treated WT mice (left panels). Scale bars = 50 μm. (d) Quantification of the number of CD68 positive macrophages in the kidney sections. Results are expressed as mean ± SE. *** p < 0.001 versus HPRT^+/+^ allopurinol treated mice. (e) Gene expression analysis of the pro-inflammatory molecules gp91phox, MCP-1 and TNF-α indicates no differences based on the genotype in mice administered vehicle (white bars), whereas a statistically significant increase of all three molecules is present in allopurinol HPRT^-/-^ kidneys. mRNA level normalized to the value of HPRT +/+ mice treated with vehicle alone Results are expressed as mean ± standard deviation. ***p< 0.001 versus mice of the same genotype treated with vehicle alone.

The yellow appearance of the kidneys of allopurinol-treated HPRT^-/-^ mice strongly suggested the presence of lipid deposits, which were confirmed by Oil Red O staining (Fig 6a). While the staining was completely negative in allopurinol-treated HPRT^+/-^ (data not shown) and WT animals, the kidneys of allopurinol-treated HPRT^-/-^ mice showed numerous red oil positive areas, mainly filling the lumen of crystal-containing tubules.

Expression analysis conducted on the mRNA extracted from the whole renal tissue showed a statistically significant activation of the C/EBPα/β pathway and a significantly decreased expression of PPARα in allopurinol-treated HPRT^-/-^ mice as compared to allopurinol-treated HPRT^+/-^ and WT mice. No changes of these molecules were observed in the kidneys of vehicle-treated animals. Moreover, no changes were observed in the expression of PPARγ among allopurinol-treated mice (Fig 6b).

### Tubulointerstitial damage

Immunostaining with the macrophage marker CD68, which was almost negative in allopurinol-treated heterozygous and WT mice as well as in all vehicle-treated animals, demonstrated numerous positive cells in the allopurinol-treated HPRT^-/-^ kidneys. Macrophages were found not only in the cortex, where they surrounded the dilated tubuli, but also in the renal medulla (Fig 6c and 6d).

Confirming the contribution of pro-inflammatory stimuli to renal damage of allopurinol-treated HPRT^-/-^ mice, significantly higher amounts of gp91phox (a subunit of NAPDH oxidase), monocyte chemoattractant protein-1 (MCP-1) and tumor necrosis factor α (TNF-α) mRNA were detected in HPRT^-/-^ kidneys than in allopurinol-treated HPRT^+/-^ and WT animals and all vehicle-treated mice (Fig 6e).

**Fig 7:**
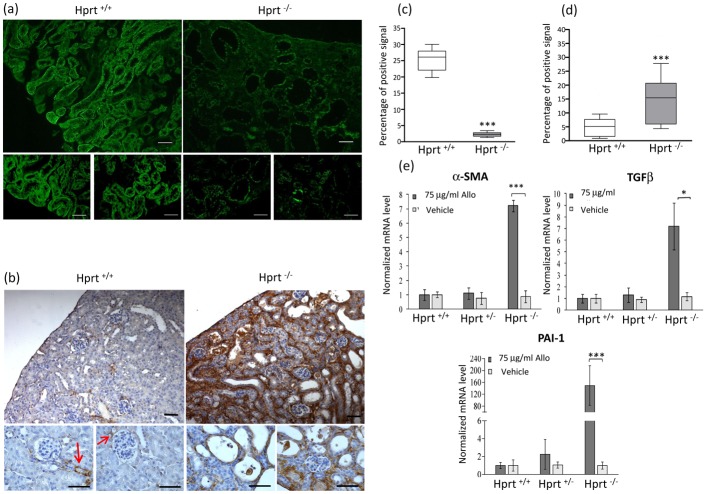
Phenotype cell changes. (a) Representative images of E-cadherin staining, showing diffuse positivity in tubular cells in kidney sections from allopurinol-treated WT mice (left panels). The right panels show corresponding tissue sections from allopurinol-treated HPRT^-/-^ animals, showing profoundly decreased E-cadherin expression in the dilated tubuli. Scale bars = 50 μm. (b) Immunohistochemical staining for α-SMA. Kidneys from allopurinol-treated WT mice (left panels) show only scattered expression of the molecule. At higher magnification (lower panels), positivity is found in vascular structures, such as peritubular capillaries and glomerular arterioles (arrows), whereas the staining is completely negative in tubules. A diffusely increased expression in the tubulointerstitium is instead observed in kidney sections from allopurinol-treated HPRT^-/-^ mice (right panels). At higher magnification, the molecule appears localized in tubular cells and around the tubules (lower panels). α-SMA, α-smooth muscle actin. Scale bars = 50 μm. Quantification of E-cadherin (c) and α-SMA (d) shows a statistically significant difference in allopurinol-treated WT and HPRT^-/-^ animals Data are presented as box plots, where the boxes represent the 25th to 75th percentiles, the lines within the boxes represent the median, and the lines outside the boxes represent the 10th and 90th percentiles. *** p < 0.001 versus WT. (e) Gene expression analysis of pro-fibrotic molecules TGF-β α-SMA and PAI-1 shows no differences based on the genotype in mice administered vehicle (white bars), whereas a statistically significant increase of all three molecules is present in allopurinol HPRT^-/-^ kidneys (gray bars). mRNA level normalized to the value of HPRT^+/+^ mice treated with vehicle alone. Results are expressed as mean ± standard deviation.*p< 0.05; *** p< 0.001 versus mice of the same genotype treated with vehicle alone.

In addition, changes of the tubular cell phenotype were observed in these animals, *i*.*e*. loss of the epithelial marker E-cadherin (Fig 7a and 7c). Correspondingly, the immunostaining for α-SMA, which was limited to the vessel wall in all vehicle-treated animals and allopurinol-treated HPRT^+/-^ and WT mice, demonstrated a diffuse tubular and peritubular positivity in allopurinol-treated HPRT^-/-^ mice (Fig 7b and 7d). These results were confirmed by mRNA changes, showing significantly increased expression of TGFβ, PAI-1, and α-SMA (Fig 7e).

### *In vitro* studies

**Fig 8:**
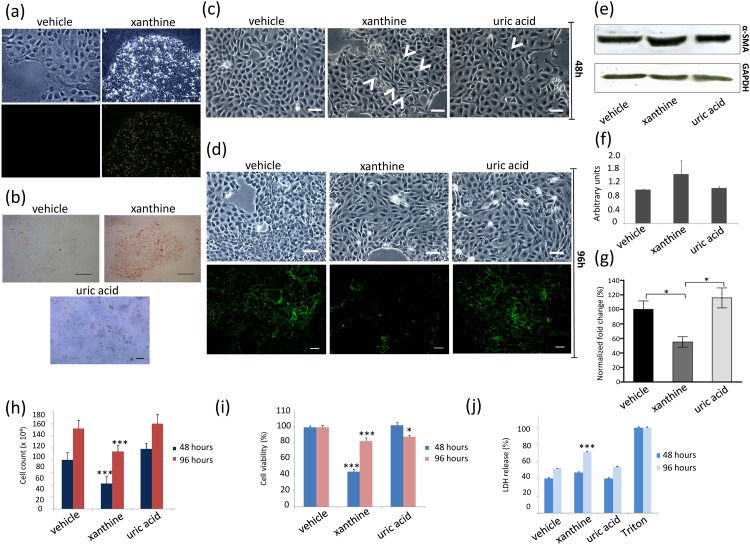
Xanthine effects *in vitro* and tubular cell phenotype. (a) Phase contrast (upper panels) and polarized microscopy (lower panels) show the deposition of xanthine after incubation for 48 h (right panels) as compared to vehicle alone (left panels). (b) Oil Red O staining demonstrates increased intracellular lipids in cells exposed to xanthine (right panel) as compared to vehicle (left panel) and uric acid (lower panel). Scale bars = 50 μm. (c) At 48 h incubation, cells exposed to vehicle (left panel) demonstrate the typical cobblestone appearance, whereas a more elongated shape (white arrows) can be observed after xanthine (middle panel) and, to a lesser extent, uric acid (right panel) incubation. Scale bars = 50 μm. (d) At 96 h incubation, light microscopy (upper panels) confirms a more diffuse appearance of elongated cells after xanthine and uric acid exposure. This associates with loss of E-cadherin expression (lower panels) particularly in xanthine-treated cells. Scale bars = 50 μm. (e) Western blot analysis and (f) densitometric quantification of α-SMA in vehicle-, xanthine- and uric acid-treated cells at 96 h. (g) Quantification results of E-cadherin staining in 96h-incubated cells. *p < 0.05 versus cells treated with vehicle alone and versus uric acid treated cells. At 96h, treatment with xanthine reduces cell number (h) and viability (i), more than vehicle and uric acid. Cytotoxicity of xanthine is confirmed by increased LDH release (j) in the medium, which is higher compared to vehicle and uric acid. The supernatant of Triton X-100-treated cells is used as the positive control. Results are expressed as mean ± SEM. *p< 0.05; ^§^ p< 0.001 versus vehicle treated cells in (h) and (i) and versus Triton treated cells in (k).

When the tubular MDCK cell line was exposed to xanthine, crystals were found diffusely deposited after 48 hours, as observed by brightfield and polarized microscopy (Fig 8a). At this time point, Oil Red O staining was diffusely positive, as compared with medium or uric acid incubation (Fig 8b). These features were accompanied by a change of morphology that was already present at 48 hours (Fig 8c) and persisted at 96 hours (Fig 8d), with elongated spindle-shaped cells instead of the typical cobblestone appearance. The effect was more diffuse than that obtained by uric acid, utilized as a positive control [13], as confirmed by a more severe loss of the epithelial marker E-cadherin (Fig 8d and 8g) and increased levels of the mesenchymal marker α-SMA (Fig 8e and 8f). Confirming the *in vivo* observed tubular damage, xanthine exposure resulted also in reduced cell viability and increased LDH release (Fig 8i and 8j), as compared to medium or uric acid exposure.

## Discussion

Our results indicate that allopurinol, when administered to mice lacking HPRT, causes early death by acute renal failure due to xanthine deposition in the renal tissue and diffuse tubulointerstitial fibrosis. The same dosages of the drug are instead not harmful to both wild type and HPRT heterozygous mice, which look healthy and have preserved renal function and completely normal kidney tissue.

The rapid development of renal failure impaired the possibility to examine any behavioral effects of allopurinol in the knockout animals, because mice were obviously suffering even at lower dosages and had to be sacrificed soon after weaning to avoid sudden death by renal insufficiency.

Xanthine is the least soluble product of purine degradation and is produced by xanthine dehydrogenase, that catalyzes the conversion of hypoxanthine to xanthine and xanthine to uric acid. The enzyme is the product of a single gene, and it is present in cells as two interconvertible forms, xanthine dehydrogenase (XDH) and xanthine oxidase (XO) [14].

In humans, xanthine urolithiasis is a rare entity, which may lead to acute renal failure[15–18], and is due to genetic or iatrogenic causes.

Inherited xanthinuria, an autosomal recessive condition [19–23] can be caused by deficiency of the (XDH), or by double deficiency of XDH and aldehyde oxidase [21, 24] or by lack of molybdenum cofactor [20], which is an essential cofactor for the function of several enzymes.

Iatrogenic xanthinuria, instead, results from treatment with allopurinol, a stereoisomer of hypoxanthine, which acts as a competitive inhibitor of XDH. This medication is used as a common treatment for gout and other hyperuricemic states. Allopurinol reduces uric acid production and increases the concentration of precursor oxypurines, which are either excreted in the urine or recycled by HPRT. Stone formation may occur in those in whom urine is supersaturated with xanthine, which is ten times less soluble in water than hypoxanthine. Patients with Lesch-Nyhan syndrome, who lack completely HPRT, or patients with partial HPRT deficiency have been reported to develop xanthine nephropathy, acute kidney failure, and stones following treatment with allopurinol [25, 26]. A few incidents of xanthine nephropathy and renal failure have been also described in patients treated with allopurinol during chemotherapy for malignancy [27]. The latter occurred either when large doses of allopurinol were used or during aggressive chemotherapy for a large tumor cell burden with concomitant allopurinol therapy.

In higher animals other than primates, xanthinuria is lethal due to kidney damage resulting from xanthine stones in the urinary tract [28–30], and this is explained by an acquired tolerance to oxypurines by primates that followed the loss of uricase during evolution [31].

Blockade of XDH by allopurinol in the HPRT-deficient mouse resulted in increased serum concentration of xanthine, hypoxanthine, and inosine. Excess of this latter metabolite may be related to the effect of increased hypoxanthine on the reversible reaction catalyzed by purine nucleoside phosphorylase [32]. The lack of oxypurine accumulation in untreated HPRT-deficient mice can be explained by the strong basal activity of mouse XDH [33]. No substantial differences in XDH expression and activity were observed between HPRT^+/+^ and HPRT^-/-^ mice (S2 Fig).

Xanthine crystals and nephropathy have been described in rats treated with XDH inhibitors [34] Mice lacking XDH were described in 2009 by Ohtsubo T and co-workers [12]. Similarly to our findings, these animals display xanthine crystals in the renal tubules, and develop renal failure due to diffuse tubulointerstitial lesions. The finding of fat rich deposition and the activation of adipogenetic pathways led the authors to suggest that absence of the enzyme, which is implicated in regulating lipid production, could be ascribed as the main culprit for these changes in gene expression. However, our data suggest a direct role for xanthine in the activation of these pathways. First, we observed renal lesions only when the inhibition of XDH by allopurinol was associated with HPRT-deficiency and serum accumulation of xanthine. Second, the addition of xanthine *in vitro* to cells with functional XDH was enough to generate tubular changes. Based on this evidence, we conclude that xanthine excess rather than XDH blockade/absence promotes the lipid deposition and the tubular damage observed in the kidneys.

Increased lipid deposition has been demonstrated to have a role in progression of renal damage by several lines of evidence; first, there are a number of genetic abnormalities of lipid metabolism directly involving the kidney, such as Fabry’s disease [35], lecithin cholesterol acyltransferase deficiency [36], genetic and acquired lipodystrophy [37]. Second, increases in serum lipids and obesity have been associated with a faster decline of renal function [38], and conversely, treatment with statins or other lipid-controlling agents protect the kidney not only by regulating systemic lipid levels, but also by directly acting on renal cells [39].

In our animals, the kidneys macroscopically showed a yellowish appearance, and Oil Red O staining was particularly intense in proximity of xanthine crystals. Around these tubular structures and around the intratubular deposits, inflammatory cells were particularly numerous, supporting the concept that crystals and lipids promote the recruitment of inflammatory cells within the interstitium.

*In vivo* and *in vitro*, xanthine caused lipid accumulation and tubular damage with rapid modification of the epithelial phenotype towards a mesenchymal pattern, with change of shape, loss of E-cadherin, and increase of α-SMA expression by tubular cells. These changes are always observed in association with progression of renal diseases, both in rodent models [40] and in humans [41], in a progressive sequence of events that represent what is currently known as partial epithelial to mesenchymal transition [42] and eventually lead to diffuse renal fibrosis and renal failure, if not promptly treated.

Thus, the results described in this work highlight the different consequences of an uricostatic treatment with allopurinol in the presence and in the absence of a functional HPRT activity. Allopurinol is the treatment of choice for both idiopathic hyperuricemia and urate overproduction due to HPRT deficiency. However, in the latter case the management of hyperuricemia is complicated by the accumulation of oxypurines and the risk of xanthine nephropathy [43]. These considerations motivate the research of other therapeutic options for the hyperuricemia induced by HPRT deficiency. Alternative urate lowering strategies that could avoid oxypurine accumulation are the inhibition of upstream enzymes in the urate biosynthetic pathway [44], and the administration of uricolytic enzymes [45, 46].

Our results also have implications for the development of a better animal model of LND. As the lack of urate degradation is a determinant of the hyperuricemic phenotype, it has been suggested that the double inactivation of HPRT and UOX genes in mouse could better reproduce the alteration of purine metabolism observed in human HPRT deficiency. Experiments are underway in our laboratories to generate double HPRT/UOX mutants by crossing single-KO mice. The UOX-KO mouse is affected by hyperuricemia and requires antenatal administration of allopurinol to avoid urate nephropathy [6]. The renal phenotype of the allopurinol-treated HPRT-deficient mouse indicates that excessive accumulation of xanthine and xanthine nephropathy must be also taken into account in the generation of the double HPRT/UOX-KO mouse.

## Supporting information

S1 Table Primer pairs used in gene expression studies. (DOCX)

S1 Fig Blood urea nitrogen and serum creatinine. (a) Blood urea nitrogen (BUN) and serum creatinine (b) display high levels in allopurinol-treated HPRT^-/-^ mice, whereas are within the normal range in HPRT^+/+^ animals. Results are expressed as mean ± standard deviation. **p < 0.01 versus HPRT^+/+^.
(TIF)

S2 Fig XO expression in kidney and serum activity in WT and HPRT mutant mice. (TIF)
