# Supplementary material for: The renal phenotype of allopurinol-treated HPRT-deficient mouse
Source: PLoS One. 2017 Mar 10;12(3):e0173512. doi: 10.1371/journal.pone.0173512 (PMC5345830; doi:10.1371/journal.pone.0173512)
Supplement: S1 Table — (DOCX) [file pone.0173512.s001.docx]

**Table S1**

| **Gene** | Primer pair | **Lenght (bp)** | **GenBank**  **assession** |
| --- | --- | --- | --- |
| **HMG-CoA** | *(F) 5'-CTTGTGGAATGCCTTGTGATTG-3'*  *(R) 5'-AGCCGAAGCAGCACATGAT-3'* | 90 | XM_006517530.1 |
| **FASn** | *(F) 5'-ACACTGCTGCGTGCCAAGAC-3'*  *(R) 5'-TCAATGATGTGCACAGACACCTTC-3'* | 105 | NM_007988.3 |
| **C/EBP-α** | *(F) 5'-TGCGCAAGAGCCGAGATAAAG-3'*  *(R) 5'-TCACGGCTCAGCTGTTCCAC-3'* | 115 | NM_006532715.1 |
| **C/EBP-β** | *(F) 5'-ACCGGGTTTCGGGACTTGA-3'*  *(R) 5'-GTTGCGTAGTCCCGTCTCCA-3'* | 71 | NM_001287738.1 |
| **PPAR-α** | *(F) 5'-TCAAGGTGTGGCCCAAGGTTA-3'*  *(R) 5'-CGAATGTTCTCAGAAGCCAGCTC-3'* | 121 | NM_011144.6 |
| **PPAR-γ** | *(F) 5'-TGTCGGTTTCAGAAGTGCCTTG-3'*  *(R) 5'-TTCAGCTGGTCGATATCACTGGAG-3'* | 122 | AB644275.1 |
| **TGF-β1** | *(F) 5'-TGCAAGACCATCGACATGGAG-3'*  *(R) 5'-GCGAGCCTTAGTTTGGACAGGA-3'* | 83 | BC013738.1 |
| **α-SMA** | *((F) 5'-ACCAACTGGGACGACATGGAA -3'*  *(R) 5'-TGTCAGCAGTGTCGGATGCTC-3'* | 90 | NM_007392.3 |
| **PAI-1** | *(F) 5'- GGACACCCTCAGCATGTTCA-3'*  *(R) 5'-TCTGATGAGTTCAGCATCCAAGAT-3'* | 91 | M33960.1 |
| **TNF-α** | *(F) 5'-AAGCCTGTAGCCCACGTCGTA-3'*  *(R) 5'-GGCACCACTAGTTGGTTGTCTTTG-3'* | 122 | NM_013693.3 |
| **MCP-1** | *(F) 5'-GCCCCACTCACCTGCTGCTACT-3'*  *(R) 5'-CCTGCTGCTGGTGATCCTCTTGT-3'* | 87 | NM_011333.3 |
| **gp91phox** | *(F) 5'-TTGGGTCAGCACTGGCTCTG-3'*  *(R) 5'-TGGCGGTGTGCAGTGCTATC-3'* | 204 | U43384.1 |
| **Xdh/XdO** | *(F) 5'-ACGCCAAACAGCTCTTCCA-3'*  *(R) 5'-CACAAGCGTTTCGGATCTTCT-3'* | 63 | NM_011723 |
| **GAPDH** | *(F) 5'-AAATGGTGAAGGTCGGTGTG -3'*  *(R) 5'-TGAAGGGGTCGTTGATGG-3'* | 108 | GU214026.1 |
