# Supplementary figures and images for: The renal phenotype of allopurinol-treated HPRT-deficient mouse
Source: PLoS One. 2017 Mar 10;12(3):e0173512. doi: 10.1371/journal.pone.0173512 (PMC5345830; doi:10.1371/journal.pone.0173512)

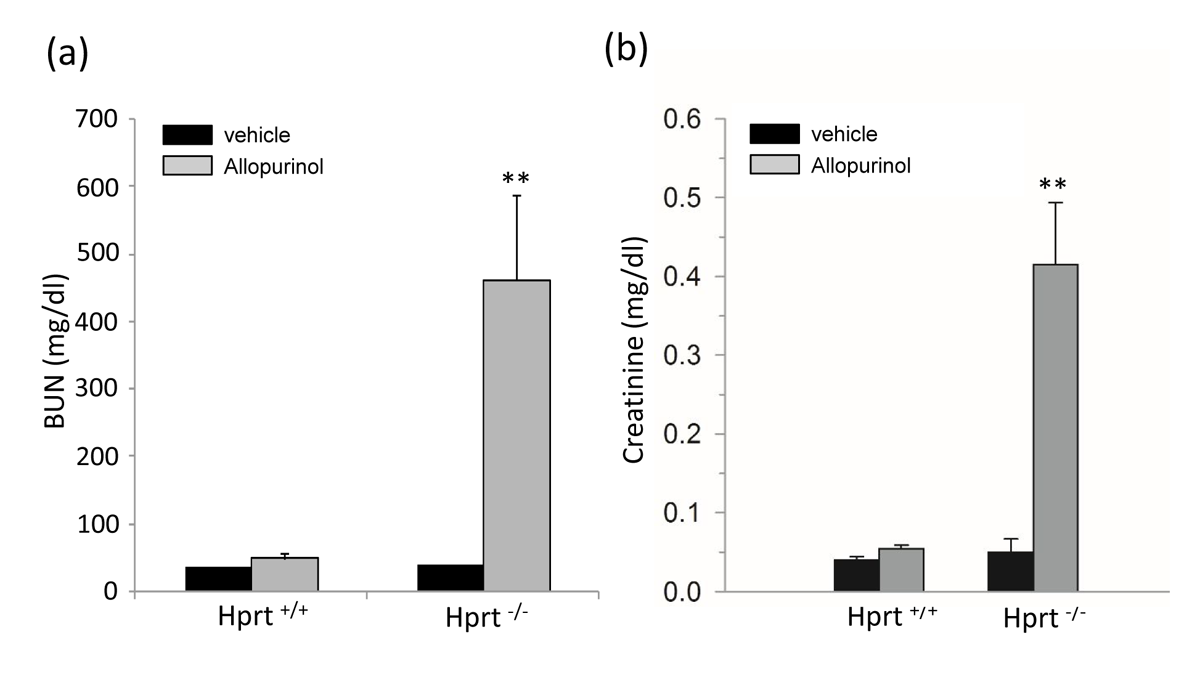

Supplement: S1 Fig — (a) Blood urea nitrogen (BUN) and serum creatinine (b) display high levels in allopurinol-treated HPRT-/- mice, whereas are within the normal range in HPRT+/+ animals. Results are expressed as mean ± standard deviation. **p < 0.01 versus HPRT+/+. (TIF) [file pone.0173512.s002.tif]

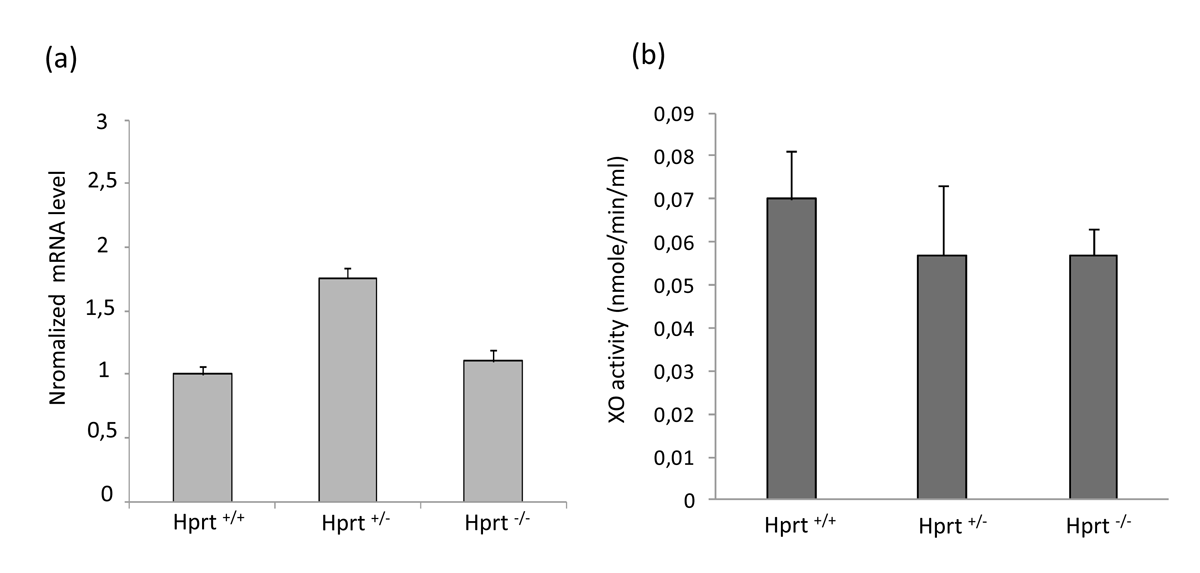

Supplement: S2 Fig — (TIF) [file pone.0173512.s003.tif]
